# Accelerated Versus Standard Ponseti Method for Idiopathic Clubfoot: A Prospective Comparative Study of Correction and Outcomes

**DOI:** 10.7759/cureus.117176

**Published:** 2026-09-29

**Authors:** JM Vyshnavi, Nagakiran K Venkatakrishnaiah, Bhuvan Tej P, DL Venkatesh, Kiran V, Uma MA

**Affiliations:** 1 Department of Orthopaedic Surgery, People's Education Society (PES) University, Kuppam, IND; 2 Department of Orthopaedic Surgery, Sri Venkateswara Medical College and Ruia Hospital, Tirupati, IND; 3 Department of Orthopaedics, People's Education Society (PES) Institute of Medical Sciences and Research, Kuppam, IND; 4 Department of Orthopaedics, Manipal Hospital, Bangalore, IND; 5 Department of Internal Medicine, People's Education Society (PES) Institute of Medical Sciences and Research, Kuppam, IND

**Keywords:** accelerated ponseti method, achilles tendon tenotomy, biweekly, congenital talipes equinovarus (ctev), foot abduction brace, foot deformity, idiopathic clubfoot, pirani scoring system, serial casting, standard ponseti method

## Abstract

Background

The standard Ponseti weekly manipulation and casting method is the gold standard for managing idiopathic clubfoot. The accelerated Ponseti biweekly manipulation and casting protocol, a promising innovation, has been proposed to significantly reduce treatment duration while maintaining efficacy. This study compared the outcomes of the accelerated and standard Ponseti techniques in terms of correction efficacy, treatment progression and the need for percutaneous Achilles tenotomy.

Methods

A prospective comparative study was conducted on 26 infants with 35 idiopathic clubfeet. Patients were allocated alternately into two groups based on their order of presentation to the clinic. The assignment was performed by the treating doctor in a strict alternating sequence. The accelerated Ponseti group (n = 17 feet) received biweekly manipulation and casting, and the standard Ponseti group (n = 18 feet) received weekly cast correction. The order of correction was the same in both groups. The Pirani score was used to determine severity and to monitor progress. The need for tenotomy was also documented. The results were analysed using appropriate statistical tests, providing a robust and comprehensive comparison of the two techniques.

Results

Baseline demographic characteristics did not differ significantly between the groups (p > 0.05). Both groups showed a progressive reduction in Pirani scores, with the accelerated group experiencing a faster decline. Tenotomy rates were comparable between the groups (p = 0.87). The accelerated Ponseti group required a significantly shorter casting duration (19.76 ± 6.9 days versus 35.39 ± 13 days; p < 0.001), indicating faster treatment completion without compromising correction.

Conclusion

The accelerated Ponseti method effectively corrects clubfoot deformities, yielding measured correction outcomes that did not significantly differ from the standard technique in this cohort. These findings indicate that the accelerated approach can significantly shorten treatment duration, providing a practical alternative for congenital talipes equinovarus (CTEV) management.

## Introduction

Congenital talipes equinovarus (CTEV), commonly known as clubfoot, is often the most prevalent congenital deformity affecting the foot and ankle, with an incidence of approximately one in every 1000 live births [1]. Studies have shown that males are affected twice as frequently as females and bilateral deformities occur in approximately 50% of cases. In unilateral cases, the right foot is more often involved [2]. Since ancient times, several ways of correcting this deformity have been documented, such as splinting, binding, casting, soft tissue releases, bony procedures and arthrodesis [3-7].

Kite first proposed a method involving repeated stretching and sequential casting to correct individual deformities. Once full correction was achieved, the Phelps splint was used to maintain alignment [8]. This technique was based on the three-point pressure principle, where manipulation involved abducting the foot under the talus while applying counterpressure over the calcaneocuboid joint. However, Ponseti later identified a critical flaw in Kite's approach, the 'Kite's error', where counterpressure at the calcaneocuboid joint restricted the abduction of the calcaneus beneath the talus. This limitation prevented the proper eversion of the calcaneum, thereby hindering the correction of heel varus [9].

The Ponseti method, developed by Ignacio Ponseti, is a nonsurgical casting technique with better outcomes than traditional surgery. It involves serial manipulation and weekly casting to gradually correct the deformity, using the talar head as the fulcrum. A percutaneous Achilles tendon tenotomy would be performed if the equinus deformity persists after correcting all other components [3,10]. Tenotomy would be followed by casting and immobilisation for at least three weeks. Later, an abduction brace would be used until the child is four to five years old [11].

The standard Ponseti casting technique protocol, with its high success rates and avoidance of extensive surgery, has established itself as the gold standard for CTEV management [11,12]. The accelerated Ponseti technique follows the same principles but uses twice-weekly cast changes, making it more convenient for patients, especially those travelling long distances for treatment. Despite the faster approach, it achieves outcomes comparable to the standard Ponseti method. Shortening the interval between casting and manipulation reduces treatment duration, minimises patient burden and improves family compliance [13,14].

The Pirani score, a reliable clinical tool, was utilised in this study to assess the severity of clubfoot deformity [15]. It evaluates six signs, divided into midfoot and hindfoot contractures, each scored from 0 to 1, aiding treatment decisions and monitoring progress in Ponseti management (the six signs are divided equally into two groups, making a maximum total score of 6 {most severe} down to 0 {fully corrected}).

The midfoot score (maximum: 3), curvature of the lateral border (CLB), evaluates if the outer edge of the foot is curved.​​​​​ Medial crease (MC) assesses the depth and prominence of creases on the inner side of the foot. Lateral head of talus (LHT) checks how easily the lateral head of the talus bone can be felt or if it covers up when manipulated.

The hindfoot score (maximum: 3), posterior crease (PC), evaluates the deep crease at the back of the ankle. Emptiness of the heel (EH) checks whether the heel cushion feels empty or high up. Rigidity of equinus (RE) measures how stiffly the ankle points downward and limits upward flexion.

Its use in this study provided a standardised and objective measure of the severity of clubfoot deformity, allowing for a comprehensive comparison of the two techniques. This study compared the clinical outcomes (primary being treatment progression and casting duration and secondary being correction efficacy and the need for percutaneous Achilles tenotomy) of the standard Ponseti and accelerated Ponseti methods for managing idiopathic congenital talipes equinovarus.

This article was recently presented as a meeting abstract at the 7th International Meet of the Asia Pacific Orthopaedic Association Young Surgeons Forum on February 22, 2025.

## Materials and methods

The study was a prospective, non-randomised comparative study conducted at a rural tertiary medical college in South India from November 2022 to December 2023. Approval was obtained from the Institutional Human Ethics Committee of People's Education Society (PES) Institute of Medical Sciences and Research (approval number PESIMSR/IHEC/101-22). The inclusion criterion included all infants diagnosed with idiopathic CTEV aged less than one year with no prior treatment for clubfoot. CTEV associated with neuromuscular or syndrome conditions were excluded from the study. Twenty-six infants with 35 idiopathic clubfeet (nine bilateral and 17 unilateral clubfeet) who met the inclusion criteria were enrolled in the study after obtaining informed consent from their parents. A prior sample size calculation was not performed; the study cohort represents a convenience sample of all eligible infants presenting to the institution during the specified timeframe

A detailed history was recorded, and a musculoskeletal assessment was done. Each foot was evaluated using the Pirani score to assess the severity of clubfoot deformity and improvements at subsequent visits [15]. Patients were allocated alternately into two groups. In Group 1, accelerated Ponseti technique subjects (n = 17 feet) received manipulation and casting twice weekly, while in Group 2, standard Ponseti technique subjects (n = 18 feet) received weekly manipulation and casting. The order of deformity correction remained consistent across both groups. The cavus was corrected first, followed by forefoot adduction, the correction of hindfoot varus and the correction of equinus deformity. If residual equinus persisted after achieving the correction of other components, a percutaneous Achilles tenotomy was performed. The foot was immobilised in a cast for at least three weeks post-tenotomy. After cast removal, a foot abduction brace was fitted for all infants. Full-time bracing (23-24 hours/day) was maintained for the first three months, followed by nighttime use (12-16 hours/day). Upon reaching walking age, boots were introduced during the day, with duration gradually increased until comfortable full-day wear was achieved.

All bilateral CTEV cases received the same casting technique, accelerated or standard Ponseti, to ensure consistency and prevent inconvenience. The primary outcome measures included the number of casts and the time required to achieve complete correction. The need for Achilles tenotomy was also noted. Subjects were followed up at regular intervals to assess compliance with the bracing protocol and recurrence rate, which is defined as any deterioration in deformity requiring re-intervention. Parents were counselled about the importance of compliance with bracing. Follow-up visits were scheduled two weeks after initiating bracing to assess adherence and every three months thereafter until the child reached five years of age. In this study, all enrolled subjects were followed up for at least six months.

Data were recorded and analysed using IBM SPSS version 20 (IBM Corp., Armonk, NY). Continuous variables were expressed as mean ± standard deviation (SD), and categorical variables were presented as percentages. Student's t-test was used to compare continuous variables, and the chi-square test was used for categorical data. A p-value of <0.05 was considered statistically significant. Intermediate Pirani scores at each cast were compared using independent t-tests to provide a descriptive tracking of the clinical trajectory, though we acknowledge that this approach does not account for repeated measures.

## Results

Thirty-five idiopathic clubfeet in 26 infants were included in the study, with 17 feet treated using the accelerated Ponseti method (Group 1) and 18 feet managed with the standard Ponseti method (Group 2).

Demographic and clinical characteristics showed that the mean age at presentation was lower in the accelerated Ponseti group (46.67 ± 30.77 days) compared to the standard Ponseti group (88.8 ± 95.4 days), though this difference was not statistically significant (p = 0.16) (Table 1). The gender distribution showed a male predominance in both groups (75.0% in Group 1 versus 71.4% in Group 2, p = 0.83). Laterality analysis revealed that bilateral involvement was more frequent in Group 1 (50%) than in Group 2 (21.4%), though this was not statistically significant (p = 0.21).

**Table 1:** Comparison of demographic and clinical characteristics between accelerated Ponseti and standard Ponseti groups This table presents the frequency, percentage and statistical comparison of baseline demographic and clinical characteristics in infants with idiopathic CTEV treated using the accelerated Ponseti or standard Ponseti method. All categorical variables are expressed as n (%), and the chi-square test is used for statistical analysis. Age, indicated by '*', is presented as mean ± standard deviation (x̄ ± s) and analysed using the t-test for statistical comparison. A p-value <0.05 is considered statistically significant CTEV, congenital talipes equinovarus; LSCS, lower-segment caesarean section

Variable	Group 1: accelerated Ponseti (n = 12 infants), n (%)	Group 2: standard Ponseti (n = 14 infants), n (%)	Statistical test	P-value
Age in days (x̄ ± s)*	46.67 ± 30.77	88.8 ± 95.4	-1.45	0.16
Gender			0.42	0.83
Female (f)	3 (25.0%)	4 (28.6%)		
Male (m)	9 (75.0%)	10 (71.4%)		
Laterality			3.14	0.21
Bilateral	6 (50.0%)	3 (21.4%)		
Left	3 (25.0%)	3 (21.4%)		
Right	3 (25.0%)	8 (57.1%)		
Mode of delivery			0.24	0.62
LSCS	4 (33.3%)	6 (42.9%)		
Normal vaginal delivery (NVD)	8 (66.7%)	8 (57.1%)		
Presentation			0.62	0.43
Breech	5 (41.7%)	8 (57.1%)		
Cephalic	7 (58.3%)	6 (42.9%)		
Birth weight (kg)			0.49	0.48
<2.4 kg (low)	5 (41.7%)	4 (28.6%)		
≥2.5 kg (normal)	7 (58.3%)	10 (71.4%)		
Parity			0.09	0.76
1 (primi)	7 (58.3%)	9 (64.3%)		
2 (multi)	5 (41.7%)	5 (35.7%)		

The mode of delivery, foetal presentation, birth weight, and maternal parity showed no significant variation between the groups (Table 1). The distribution of infants born via lower-segment caesarean section (LSCS) and normal vaginal delivery (NVD) was nearly identical in both groups (p = 0.62). Likewise, the incidence of breech presentation was comparable (p = 0.43), and the presence of low birth weight did not show a significant difference (p = 0.48).

At presentation, the accelerated Ponseti group demonstrated a higher mean Pirani Midfoot Contracture Score (MFCS) than the standard group (p = 0.020), while independent t-tests showed that this difference persisted after the first cast (p = 0.037). Similarly, after the first cast, the MFCS remained significantly higher in the accelerated group (2.06 ± 0.429 versus 1.56 ± 0.856, p = 0.037). By the fifth cast, the scores were nearly identical. No other significant differences were observed in Hindfoot Contracture Score (HFCS) or total Pirani scores at any point (Table 2). Both groups showed consistent improvement in Pirani scores with successive casts (Figure 1). By the fifth cast, the scores were nearly identical. By the end of the casting period, both groups achieved near-complete correction, with no significant differences in the final Pirani scores. The need for percutaneous tenotomy was similar between groups (52.9% versus 55.6%, p = 0.870). Notably, the accelerated Ponseti group required a significantly shorter casting duration (19.76 ± 6.99 days versus 35.39 ± 13.03 days, p < 0.001), indicating faster treatment completion without compromising correction (Table 2). Within the limited early follow-up period (a minimum of six months for all subjects), no immediate clinical recurrences were observed. However, this early timeframe is insufficient to evaluate true long-term recurrence rates.

**Table 2:** Comparison of Pirani scores between accelerated and standard Ponseti methods The table compares the accelerated Ponseti and standard Ponseti groups based on Midfoot Contracture Score (MFCS), Hindfoot Contracture Score (HFCS) and total Pirani scores at various treatment stages. It also analyses the total number of casts, casting duration and the need for percutaneous tenotomy. The analysis includes 35 feet, with bilateral cases considered as two separate feet. Note: because feet achieved full correction at different stages (mean total casts: 5.65 in Group 1 and 5.06 in Group 2), the denominator of active feet decreased at the sixth and seventh casting stages. Feet that achieved complete correction prior to these later stages exited the active casting protocol. Continuous variables, denoted by '*', are expressed as mean ± standard deviation (x̄ ± s) and analysed using the t-test for statistical comparison. Percutaneous tenotomy, being a categorical variable, is presented as n (%), and the chi-square test is used for statistical analysis *A p-value of less than 0.05 is considered statistically significant

Variables		Group 1 (n = 17 feet), accelerated Ponseti (x̄ ± s)	Group 2 (n = 18 feet), standard Ponseti (x̄ ± s)	Statistical test	P-value
At presentation*	Pirani MFCS	2.18 ± 0.52	1.67 ± 0.68	2.45	0.02*
	Pirani HFCS	2.00 ± 0.86	1.89 ± 0.32	0.50	0.61
	Total Pirani score	4.24 ± 1.25	3.94 ± 1.25	0.68	0.49
After the first cast*	Pirani MFCS	2.06 ± 0.42	1.56 ± 0.85	2.17	0.03*
	Pirani HFCS	1.88 ± 0.78	1.78 ± 0.64	0.43	0.66
	Total Pirani score	3.94 ± 1.02	3.44 ± 1.09	1.38	0.17
After the second cast*	Pirani MFCS	1.76 ± 0.56	1.56 ± 0.85	0.84	0.40
	Pirani HFCS	1.88 ± 0.78	1.67 ± 0.68	0.86	0.39
	Total Pirani score	3.82 ± 1.13	3.22 ± 1.51	1.32	0.19
After the third cast*	Pirani MFCS	1.35 ± 0.78	1.44 ± 0.78	-0.34	0.73
	Pirani HFCS	1.76 ± 0.83	1.56 ± 0.85	0.73	0.46
	Total Pirani score	2.76 ± 1.56	2.89 ± 1.53	-0.23	0.81
After the fourth cast*	Pirani MFCS	0.82 ± 0.95	0.56 ± 0.70	0.95	0.34
	Pirani HFCS	1.53 ± 0.74	1.53 ± 0.80	0.01	0.98
	Total Pirani score	2.00 ± 1.32	1.67 ± 0.97	0.85	0.40
After the fifth cast*	Pirani MFCS	0.35 ± 0.49	0.39 ± 0.50	-0.21	0.83
	Pirani HFCS	0.82 ± 0.80	0.89 ± 0.96	-0.21	0.83
	Total Pirani score	1.18 ± 1.01	0.94 ± 0.99	0.68	0.50
After the sixth cast*	Pirani MFCS	0.00 ± 0.00	0.00 ± 0.00	-	-
	Pirani HFCS	0.47 ± 0.87	0.78 ± 1.00	-0.96	0.34
	Total Pirani score	0.59 ± 0.87	0.78 ± 1.00	-0.59	0.55
After the seventh cast*	Pirani MFCS	0.00 ± 0.00	0.00 ± 0.00	-	-
	Pirani HFCS	0.24 ± 0.43	0.67 ± 0.90	-1.77	0.08
	Total Pirani score	0.41 ± 0.79	0.78 ± 1.00	-1.19	0.24
Percutaneous tenotomy, n (%)	No	8 (47.1%)	8 (44.4%)	0.024	0.87
	Yes	9 (52.9%)	10 (55.6%)		
Total number of casts*		5.65 ± 1.99	5.06 ± 1.86	0.90	0.37
Total duration of cast treatment		19.76 ± 6.99	35.39 ± 13.03	-4.38	0.000

**Figure 1:**
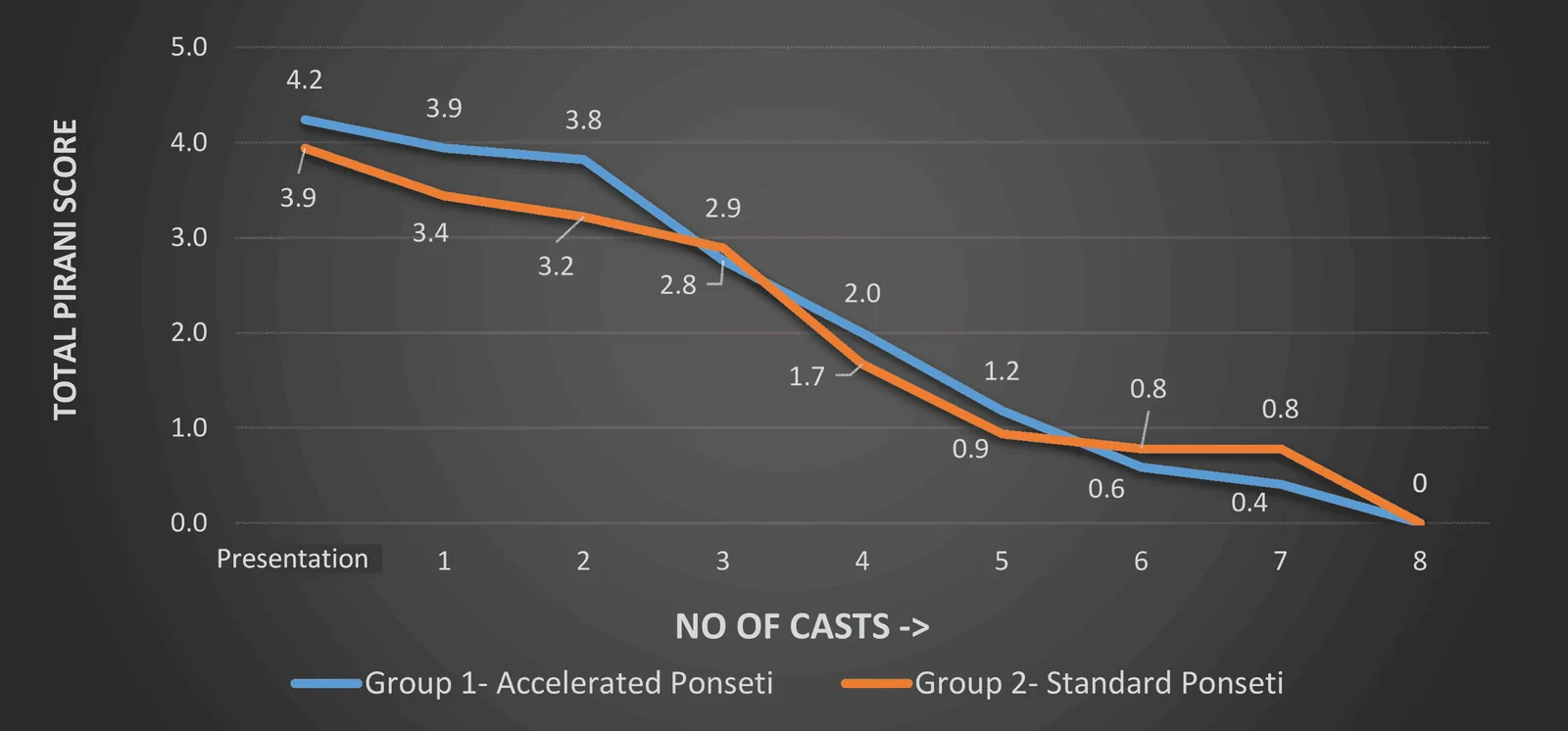
Change in the mean Pirani score over subsequent casts in accelerated Ponseti versus standard Ponseti methods This graph illustrates the change in the total Pirani score over subsequent casts for CTEV treated with accelerated Ponseti and standard Ponseti. In the accelerated Ponseti method, cast changes are performed twice a week, leading to a potentially faster correction than the standard Ponseti method, where casts are typically changed weekly. The number of active feet represented in the later casting stages (casts 5-7) progressively decreases as individual feet achieved full clinical correction and completed the casting phase CTEV: congenital talipes equinovarus

Correlation analysis showed a statistically significant moderate positive correlation between age and the total duration of cast treatment (r = 0.320; p = 0.030), indicating that older infants required a more extended casting period (Table 3). Similarly, a significant moderate positive correlation was observed between age and the total Pirani score at presentation (r = 0.424; p = 0.006), suggesting that older infants tended to present with more severe deformities. However, no significant correlation was found between age and the total number of casts required (r = 0.121; p = 0.244).

**Table 3:** Correlation of age at presentation with other variables This table presents the correlation between age at presentation and key variables. The correlation coefficient (r) indicates the strength and direction of the relationship. A positive value suggests that the respective variable tends to increase as age increases *A statistically significant correlation (p < 0.05)

Variable	Correlation (r)	P-value
Total duration of cast treatment	0.32	0.03*
Total number of casts	0.12	0.24
Total Pirani score at presentation	0.42	0.006*

Correlation analysis between the total Pirani score at presentation and the duration of cast treatment showed a moderate positive correlation (r = 0.43; p = 0.010), suggesting that higher initial Pirani scores were associated with a more extended casting period (Table 4). Similarly, a strong positive correlation was observed with the total casts required (r = 0.72; p < 0.001), indicating that infants with higher Pirani scores generally needed more casts for correction.

**Table 4:** Correlation of total Pirani score at presentation with the duration of cast treatment and the total number of casts This table shows the correlation between the total Pirani score and key parameters at presentation. The correlation coefficient quantifies the strength and direction of the relationship *A statistically significant correlation (p < 0.05)

Variables	Correlation coefficient	P-value
Total duration of cast treatment	0.43	0.010*
Total number of casts	0.72	0.000*

## Discussion

Clubfoot presents a complex challenge that tests the expertise and proficiency of orthopaedic surgeons [16]. The Ignacio Ponseti casting protocol is based on the pathoanatomy and fundamental kinematics of the deformity [17]. This method effectively realigns clubfoot in infants without extensive surgical interventions by leveraging the biomechanical properties of fibroelastic connective tissue to correct the deformity.

Gender distribution

This study observed a male-to-female ratio of 2.7:1, consistent with existing literature reporting a higher prevalence of clubfoot in males. No significant difference was found between the accelerated Ponseti and standard Ponseti groups regarding gender distribution (p > 0.05). Previous studies have reported a male-to-female ratio of around 5:1 in infants with idiopathic clubfoot [18]. Evans et al. observed a male-to-female ratio of approximately 2.5:1 [19].

Laterality

In our study, bilateral cases accounted for approximately 51.5% of the total, with right-sided foot deformity (31.4%) higher than left-sided cases (17.1%) among unilateral CTEV. Salvatori et al. observed bilaterality in 43.3% of cases [18]. Evans et al. reported bilaterality in 54%, with left-foot and right-foot involvement in 18% and 28% of cases, respectively [19]. No significant difference in laterality distribution was found between Group 1 and Group 2 (p > 0.05).

Age at presentation

Most subjects in this study were between one and six months of age, with the youngest being four days old and the oldest 12 months. There was no significant difference in the age of presentation between Group 1 and Group 2 (p > 0.05). Correlation analysis indicated that the age at the initiation of treatment might influence the severity of the deformity and the duration of treatment (Table 3). Early intervention remains crucial, as starting treatment within the first month of life has been associated with better outcomes [20].

It is important to interpret our findings in the context of baseline imbalances between the two cohorts. Although the difference in mean age at presentation was not statistically significant, the standard Ponseti group presented, on average, nearly 40 days older than the accelerated group. Furthermore, the accelerated group presented with a significantly higher initial Midfoot Contracture Score (MFCS). This indicates that the accelerated cohort had a younger but more severe initial midfoot presentation. The fact that the accelerated group still achieved correction in significantly fewer days, despite the more severe baseline midfoot deformity, is clinically encouraging. However, the lack of exact baseline matching remains a confounding factor.

Pirani score

At presentation, the accelerated group had a higher mean Pirani Midfoot Contracture Score (MFCS) (2.18 ± 0.52) compared to the standard group (1.67 ± 0.68; p = 0.020), indicating a more severe initial midfoot deformity (Table 2). However, the groups had no significant differences in the Hindfoot Contracture Score (HFCS) or total Pirani score. Both groups demonstrated progressive deformity correction with successive casts, with substantial differences observed only after the first cast (p = 0.03). Correlation analysis revealed a significant relationship between the severity of clubfoot deformity at presentation and the subsequent course of treatment (Table 4). This finding highlights the importance of early detection and intervention [19,20].

Number of casts required and treatment duration

A comparison between Group 1 and Group 2 showed no significant difference in correction rates (p > 0.05). Both groups required an average of five casts for correction, consistent with findings from other studies reporting an average of five casts [19]. The accelerated group had a significantly shorter treatment duration (19.76 ± 6.99 days versus 35.39 ± 13.03 days; p < 0.001) without additional surgery. The tenotomy rates were comparable between the groups (52.9% versus 55.6%; p = 0.87). Observations of the study align with previous findings showing median treatment durations of 16 days for the accelerated group compared to 42 days for the standard Ponseti group (p < 0.001) [19,21,22].

Pirani score and number of casts required

Lower initial Pirani scores (3-4) were associated with faster correction, whereas higher scores (5-6) indicated more rigid deformities, requiring more casts. In this study, clubfeet with initial Pirani scores between 4.5 and 6 required an average of 5.7 casts for correction, whereas those with scores below 4.5 required an average of 3.1 casts. Correlation analysis between the total Pirani score at presentation and the duration of cast treatment showed a moderate positive correlation (r = 0.43; p = 0.010), suggesting that higher initial Pirani scores were associated with a more extended casting period (Table 4). Similarly, a strong positive correlation was observed with the total casts required (r = 0.72; p < 0.001), indicating that infants with higher Pirani scores generally needed more casts for correction. These findings align with existing literature, reinforcing the relationship between initial severity and treatment duration [22].

Tenotomy

In this study, 54.3% of feet required percutaneous tenotomy of the Achilles tendon, with a slightly lower rate observed in Group 1 than in Group 2. However, the difference was not statistically significant (p > 0.05). Previous studies have estimated that approximately 80% of clubfoot cases treated with the Ponseti technique require tenotomy [20,23]. The lower rate in this study may be attributed to early patient presentation and shorter casting intervals.

Overall, there were no statistically significant differences between the accelerated Ponseti and standard Ponseti regarding gender incidence, laterality, age at presentation, the number of casts required, Pirani scores or tenotomy rates (p > 0.05 for all parameters). However, the accelerated Ponseti protocol significantly reduced the treatment duration without increasing tenotomy rates. These findings are consistent with existing literature, underscoring the importance of early intervention and consistent follow-up in optimising clubfoot management outcomes [17-23].

Early observations at a minimum of six months post-correction showed no immediate relapses in either group. Clubfoot recurrence frequently occurs well beyond this initial timeframe and remains heavily dependent on long-term foot abduction brace compliance. Because exact long-term follow-up durations were not evaluated in this initial cohort analysis, this study cannot establish long-term equivalence regarding recurrence rates or the permanent prevention of relapse between the accelerated and standard methods.

Limitations of the study

Sample Size and Power

The lack of an a priori power calculation and the small cohort (26 infants) increase the risk of a type II error, limiting the detection of subtle but clinically meaningful differences in tenotomy or recurrence rates.

Study Design

The non-randomised, single-centre approach introduces selection bias and limits external validity, as outcomes may reflect institutional protocols, patient demographics or specific surgeon expertise rather than the broader population.

Statistical Methodology

Relying on separate independent t-tests instead of a repeated-measures model fails to capture longitudinal trajectories and increases the risk of a type I error.

Sparse cell counts for categorical variables were analysed without Fisher's exact test, violating chi-square assumptions.

Bilateral feet from the same patient were analysed without statistical clustering, potentially underestimating standard errors.

Follow-Up Duration

The early follow-up timeframe is insufficient to draw conclusions regarding long-term recurrence or final functional outcomes, which typically emerge beyond the first year and depend heavily on bracing compliance.

Future Directions

Multicentre, randomised controlled trials with larger sample sizes are required to validate these comparative findings.

## Conclusions

The accelerated Ponseti method, compared to the standard method, effectively reduces treatment duration for idiopathic clubfoot without increasing the need for surgical interventions. It achieves comparable deformity correction in a shorter timeframe, making it a viable, time-efficient alternative, especially in resource-limited settings. The shorter treatment duration can increase patient and family compliance, as families may prefer a shorter treatment period.
